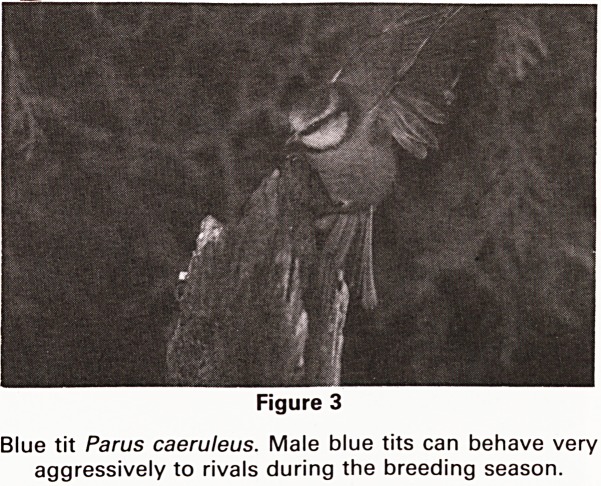# Distraction, Displacement and Alarm

**Published:** 1986-12

**Authors:** Philip Radford


### Distraction, Displacement and Alarm

**By Philip Radford**

As I was watching a lapwing *Vanellus vanellus* sitting on hard-set eggs on a piece of rough pasture, a man leading a terrier came along. Through my binoculars, I saw the lapwing becoming increasingly anxious and, when the intruders were about 200 metres away. the bird ran off to trail a wing in front of them. The restrained dog clearly wanted to chase the injury-feigning bird and, had it been allowed to do so, would have been led well away from the nest. The lapwing's distraction would almost certainly have been protective for the clutch; following the incident, incubation was placidly resumed.

A grazing sheep then ambled towards the nest. This time the lapwing did not move off the eggs until the sheep was at a distance of two metres, when the broken wing trick was repeated. Not surprisingly, the sheep made no attempt to follow but, instead, walked closer to the nest when, as though sensing failure, the bird rose in the air and dived, screaming, at the unwelcome animal. The lapwing's mate joined in the attack and the combined swooping diverted the sheep away from the eggs: interestingly, this tactic was not used until the nest was almost trampled on. Unlike the man and his dog, the sheep was not regarded as an enemy until it came very close to the lapwings eggs.

Normally it is ground-nesting birds which feign injury rather than those which build in trees or shrubs. Game birds such as pheasants *Phasianus colchicus* commonly behave in this way: here, the eggs hatch together and the young quickly disperse, being carefully tended by the hen. The well-camouflaged chicks will crouch in cover as the adult tries to lure off a fox *Vulpes vulpes* or a buzzard *Buteo buteo* to a safe distance.

**Figure 1**

Wood warbler *Phylloscopus sibilatrix*. Juvenile about to leave nest. If young are threatened, the parent bird will carry out a distraction display.

Furthermore, small moorland birds which build in grass or heather will flutter away from the nest if disturbed; examples are the skylark *Alauda arvensis* and the meadow pipit *Anthus pratensis*. Amongst warblers, the willow warbler *Phylloscopus trochilus* and the wood warbler *P. sibilatrix* both nest on the ground in wooded areas and each will progress with spread tail, and dragging a wing, if young are threatened. Turning to dunes or shingle expanses, the ringed plover *Charadrius hiaticula* is a common wing trailer and so is the oystercatcher *Haematopus ostralegus*, especially if chicks are in hiding. As with lapwings, a pair of either of these waders will demonstrate violently at an enemy in their breeding territories by swooping and calling aggressively.

But birds may distract in other ways if predators appear. This was shown to me one April day when I was admiring purple sandpipers *Calidris maritima* feeding on weed-covered tidal rocks on the Cornish coast. Suddenly a stray dog raced towards the wader group when all but one of the birds took flight; the remaining one crouched low and ran towards the sea, looking very much like a rat *Rattus rattus*. The purple sandpipers were on their way north to breed on the Arctic tundra; there, an adult will entice a fox away from its young in this manner. Nevertheless, I did not expect to see this behaviour, a 'rodent-run', from one of a migratory flock.

Probably distraction behaviour with birds indicates a conflict between fear and the drive to look after chicks or eggs. Injury-feigning does not appear in tame or semi-tame birds as a reaction to man's presence: this must depend on recognition of man as an animal – in this case, harmless. Of course, people may themselves show distraction behaviour when seeking attention and sympathy. A person with a hysterical limb paralysis is revealing his mental problems and conflict; as another illustration, symptoms may become exaggerated in an attempt to obtain financial compensation for an injury, giving rise to a traumatic neurosis.

Distraction displays by birds are necessarily conspicuous but so are the purposeless acts sometimes carried out when they are frustrated. For instance, one spring day I saw two robins *Erithacus rubecula* posturing at each other by swaying from side to side and puffing the red breast; eventually, one robin gave way and, turning aside, it pecked vigorously at the ground. Doubtless this earth pecking was a re-direction of its energy, after failing to drive the other robin away. Again, as I spotted two cock blackbirds *Turdus merula* disputing over an earthworm, one of them unexpectedly flew off with the prize; the defeated bird at once pecked into the earth in apparent anger. In the same way, it is not uncommon for a starling *Sturnus vulgaris* to preen, quite unnecessarily from the point of view of its plumage, on losing a tussle with another bird over a food scrap or, perhaps, having been prevented from entering a pool to bathe. Moreover, some incubating birds, such as herring gulls *Larus argentatus*, will add materials to the nest after a flight: pent-up energy has to be released.

With human beings, similar displacement activities are commonplace. A person who cannot get his own way at a committee meeting may bang the table in frustration or a thwarted child might kick out at the furniture, thus showing his angry feelings. But a recent observation showed a different type of behaviour by a male blackbird when a young bird, possibly its own well-grown chick, was killed by a passing car. The blackbird boldly mounted and tried to mate with the dead juvenile in a suburban street; presumably, it was the submissive attitude which led to the sexual advance.

Then people who have escaped from a near-disaster may just roar with laughter or weep copiously. Such reactions may relieve mental stress but, otherwise, are quite inappropriate. Again, with birds, a male sedge warbler *Acrocephalus schoenobaenus* will often sing energetically from cover if it is disturbed by nearby shooting or by stone throwing; it is difficult to suggest what useful purpose is served by this form of singing. Perhaps it should be remembered that, if tense, a person

**Figure 2**

Robin *Erithacus rubecula*. If frustrated, a robin may peck at the ground as a displacement activity.

may comb the hair repeatedly, bite the finger nails or chew the end of a pencil – all unnecessary actions. As further examples, I saw a chaffinch *Fringilla coelebs* whose nest had been robbed by a magpie *Pica pica* preen itself with vigour and, after a fight with a rival, a victorious cock blackbird picked up and waved a dead leaf in its bill. Not infrequently, displacement bathing may take place after conflict; the movements of bathing are carried out but not in water although, normally, there is water within sight.

It is not always suitable for birds, especially those species which do not nest on the ground, to use distraction displays inviting an enemy's attention. Alarm calling and mobbing may be more helpful here, as when a roosting tawny owl *Strix aluco* is discovered by small birds by day. This is advertisement of the situation by sound and will bring in other birds for support or, equally, may attract a hungry bird-of-prey; nestlings, responding to the parents' acoustic signals, become quite immobile. Occasionally, such blatant alarm proves annoying to the observer; I recall a wren *Troglodytes troglodytes* which called noisily when it discovered me sitting under a bush, watching red deer *Cervus elaphus* grazing peacefully not far away. Although I had taken care that the deer would not receive my scent, they suddenly became suspicious and, sighting me, bounded off at speed; undoubtedly, the wren's alarm had been the alerting signal. The wren, in fact, was feeding young nearby and, in compensation, I had close-up views of its family.

Now a householder threatened by a burglar may well scream and, similarly, a woman whose bag is snatched by a thief in the street might scream as well. Clearly, a loud noise may scare off a timid aggressor as well as drawing attention to a possible crime; hopefully, other people will be attracted to render aid. Alternatively, a potentially dangerous situation can induce complete inactivity, even stupor, in some individuals; probably they will show sweating, dilated pupils and a rapid heart action yet are unable to react positively to avert the hazard. Should this happen in the wild, the outlook for survival must be poor. But at times this does occur in nature. As an example, a frightened bird can become inert and allow itself to be picked up; in a short time, however, it will recover and run or fly off. I remember a trapped male redstart *Phoenicurus phoenicurus* which became apparently unconscious but, after two or three minutes, it flew away strongly. Presumably, syncope through vagal nerve inhibition is a likely cause of this inert state under such circumstances.

Occasionally, a particular posture may be adopted as a reaction to a fright. Once I was watching a wren feeding in a hedge when a sudden explosive noise occurred; immediately, the wren assumed a stiff, upright posture with the beak pointing vertically upwards. This cryptic position, typically like that of a bittern *Botaurus stellaris,* was maintained for several seconds before the bird gradually relaxed and again started searching for food items. Returning to people, a common remark is 'I was scared stiff' as a response to a frightening episode or someone may state that he became 'rooted to the spot' or even 'petrified'. The reactions of 'shell-shocked' soldiers as noted during the First World War must have been similar in certain instances.

Of course, loud noise or cries can be intimidatory – hence the use of 'war cries' by warriors in all parts of the world. As an illustration, the Genoese crossbowmen with the French forces at the battle of Crécy in 1346 employed loud shouting before each of their attacks on the English. Nevertheless, in this case, it seems that the stratagem had little effect! Even so, in general, a person who is trying to assert himself or to domineer in a group often adopts a louder voice.

In contrast with the protesting calls of a mobbing bird, there is the silent bolt for cover when a predatory bird is on the wing. Hence, a chaffinch will flee from a flying sparrowhawk *Accipter nisus* after giving, or hearing, a whistle which is most difficult to localise. This flight to safety is easily overlooked by people, even some naturalists, but the action, together with the sound signal which came before, is often lifesaving for the bird. Should the sparrowhawk perch on a branch, then small birds will gradually emerge from their leafy cover and, although still frightened, will begin alarm calling; for this mobbing, loud and readily localized notes are utilised. With the hawk perched, there is no immediate danger for the finch but once the predator is in the air the situation is entirely altered.

**Figure 3**

Blue tit *Parus caeruleus*. Male blue tits can behave very aggressively to rivals during the breeding season.

Whether a predatory bird is seeking a victim or rival cocks are attempting to establish a territory in spring, both circumstances require the full concentration of the birds concerned and conflict in any form involves considerable energy usage. I remember two blue tits *Parus caeruleus* fighting near a nest-hole in springtime; so intent were the birds on their aggression that I was able to separate them physically and, indeed, by the time I found them they were almost exhausted. As birds expend so much muscular and, presumably, mental energy, it is hardly surprising that distraction and displacement behaviour is part of their way of life. The

*(continued on page 148)*

### Distraction, Displacement and Alarm. *(continued from page 145)*

trailing wing, fanned tail or distorted body all draw attention to the parent bird and must mean increased danger for it but, as a result, there is an increased chance of survival for the young. There must be added risk also when a bird is engaged in, say, ground pecking or displacement preening: certainly, bird psychology is no simple matter!

If bird psychology presents many problems, then the displacements, distractions and fear reactions of man are still more difficult to interpret. The bird's brain, featuring a well-developed corpus striatum, has evolved to control the specialised life of the animal; as might be expected, there are extensive nervous connections between the corpus striatum and the mid-brain and cerebellum. The cerebellum is relatively large in all birds and doubtless has an important part to play in the control and co-ordination of flight, landings and pecking movements. The human brain is characterised by extensive development of the cerebral cortex and frontal lobes or, at any rate, those parts of the nervous system which are concerned with reasoned thought. Sadly, man does not show great success in helping the neuroses and frustrations of affected society and, moreover, aggression and associated alarm signals are all part of modern community living. If biologists and naturalists can advance their psychological analysis of the behaviour of birds then, maybe, there would be increased hope for the understanding of human mental conflict and related problems.

## Figures and Tables

**Figure 1 f1:**
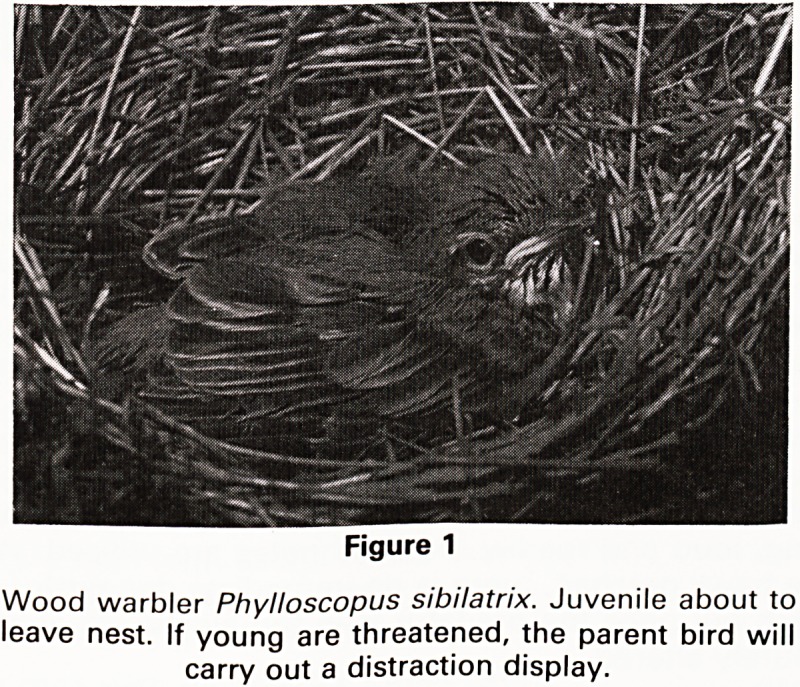


**Figure 2 f2:**
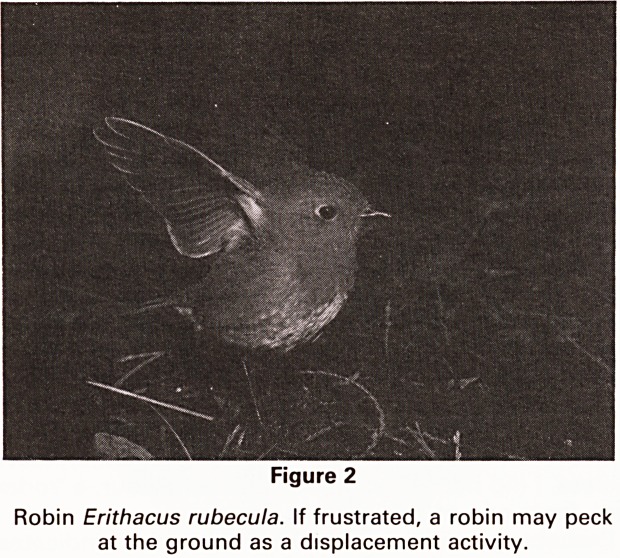


**Figure 3 f3:**